# Anti-rituximab antibodies demonstrate neutralizing capacity, associate with lower circulating drug levels and earlier relapse in lupus

**DOI:** 10.1093/rheumatology/keac608

**Published:** 2022-11-12

**Authors:** Chris Wincup, Nicky Dunn, Caroline Ruetsch-Chelli, Ali Manouchehrinia, Nastya Kharlamova, Meena Naja, Barbara Seitz-Polski, David A Isenberg, Anna Fogdell-Hahn, Coziana Ciurtin, Elizabeth C Jury

**Affiliations:** Centre for Rheumatology Research, Division of Medicine, University College London, London, UK; Department of Clinical Neuroscience, Karolinska Institutet, Stockholm, Sweden; Center for Molecular Medicine, Karolinska University Hospital, Stockholm, Sweden; Laboratoire d’Immunologie, CHU de Nice, Université Côte d’Azur, Nice, France; Centre Méditerranéen de Médecine Moléculaire (C3M), INSERM U1065, Université Côte d’Azur, Nice, France; Unité de Recherche Clinique Côte d’Azur (UR2CA), Université Côte d’Azur, Nice, France; Department of Clinical Neuroscience, Karolinska Institutet, Stockholm, Sweden; Center for Molecular Medicine, Karolinska University Hospital, Stockholm, Sweden; Department of Clinical Neuroscience, Karolinska Institutet, Stockholm, Sweden; Center for Molecular Medicine, Karolinska University Hospital, Stockholm, Sweden; Centre for Adolescent Rheumatology Research, Division of Medicine, University College London, London, UK; Laboratoire d’Immunologie, CHU de Nice, Université Côte d’Azur, Nice, France; Unité de Recherche Clinique Côte d’Azur (UR2CA), Université Côte d’Azur, Nice, France; Centre for Rheumatology Research, Division of Medicine, University College London, London, UK; Department of Clinical Neuroscience, Karolinska Institutet, Stockholm, Sweden; Center for Molecular Medicine, Karolinska University Hospital, Stockholm, Sweden; Centre for Rheumatology Research, Division of Medicine, University College London, London, UK; Centre for Adolescent Rheumatology Research, Division of Medicine, University College London, London, UK; Centre for Rheumatology Research, Division of Medicine, University College London, London, UK

**Keywords:** SLE, B cells, immunological techniques, biological therapy, autoantigens and autoantibodies

## Abstract

**Objectives:**

High rates of anti-drug antibodies (ADA) to rituximab have been demonstrated in patients undergoing treatment for SLE. However, little is known with regard to their long-term dynamics, impact on drug kinetics and subsequent implications for treatment response. In this study, we aimed to evaluate ADA persistence over time, impact on circulating drug levels, assess clinical outcomes and whether they are capable of neutralizing rituximab.

**Methods:**

Patients with SLE undergoing treatment with rituximab were recruited to this study (*n* = 35). Serum samples were collected across a follow-up period of 36 months following treatment (*n* = 114). Clinical and laboratory data were collected pre-treatment and throughout follow-up. ADA were detected via electrochemiluminescent immunoassays. A complement dependent cytotoxicity assay was used to determine neutralizing capacity of ADA in a sub-cohort of positive samples (*n* = 38).

**Results:**

ADA persisted over the 36-month study period in 64.3% of patients undergoing treatment and titres peaked earlier and remained higher in those who had previously been treated with rituximab when compared with than those who were previously treatment naive. ADA-positive samples had a significantly lower median drug level until six months post rituximab infusion (*P* = 0.0018). Patients with persistent ADA positivity showed a significant early improvement in disease activity followed by increased rates of relapse. *In vitro* analysis confirmed the neutralizing capacity of ADA to rituximab.

**Conclusions:**

ADA to rituximab were common and persisted over the 36-month period of this study. They associated with earlier drug elimination, an increased rate of relapse and demonstrated neutralizing capacity *in vitro*.

## Graphical Abstract

**Figure:**
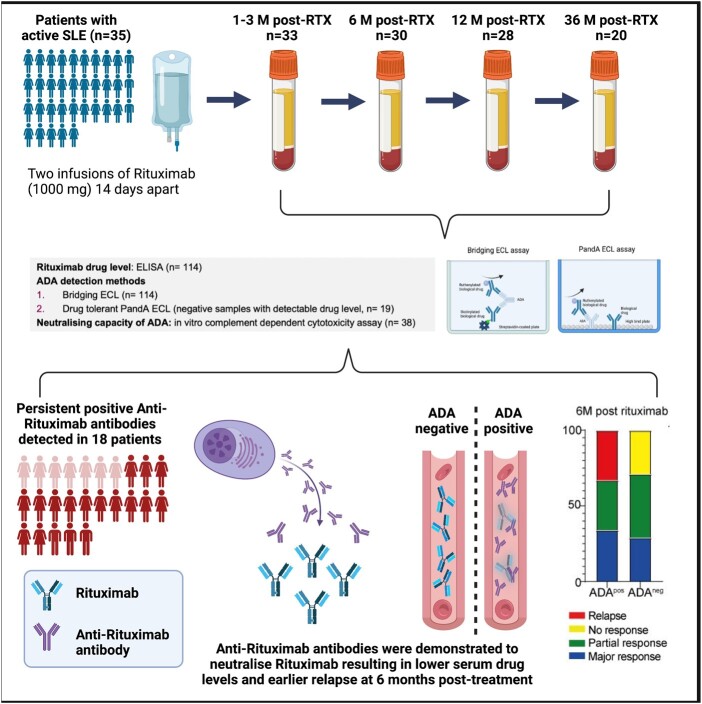

Rheumatology key messages

- A large proportion of patients with SLE (64.3%) treated with rituximab develop persisting anti-drug antibodies.
- Patients with anti-drug antibodies to rituximab show lower drug levels and earlier disease relapse.
- ADA have a neutralizing capacity suggesting clearance of rituximab by ADA may result in relapse.

## Introduction

Anti-drug antibodies (ADA) to rituximab have been reported in 4–11% of patients undergoing treatment for RA [1] and in 26–37% of those receiving treatment for multiple sclerosis (MS), where they associate with impaired B-cell depletion and variable clinical response [2]. In spite of not reaching primary endpoints in two large randomized controlled trials [3, 4], rituximab has been demonstrated to be effective in the treatment of severe and refractory SLE in a number of open label studies [5–7]. Of note, ADA to rituximab have previously been found at increased frequency in patients with SLE compared with RA [1], MS [2] and vasculitis [8]. In a previous cross-sectional study, we demonstrated that ADA to rituximab were associated with infusion-related reactions [9]; however, the long-term implications of ADA to rituximab in SLE are poorly understood. Furthermore, to date, little is known about whether these antibodies contain neutralizing capability that may impair response to treatment.

The aim of this study was to evaluate the dynamics of ADA to rituximab over time and assess the long-term impact on both immunological and clinical outcomes. In addition, we sought to study the impact of ADA on circulating drug levels and investigate neutralizing capacity *in vitro*.

## Methods

### Patients

All patients recruited to this retrospective observational study underwent treatment with rituximab (Mabthera, Roche, Basel, Switzerland) for active SLE at University College London Hospital (UCLH) between 2002 and 2016 (*n* = 35). Treatment consisted of two 1000 mg intravenous doses of rituximab over 6–8 h on day 1 and day 14. Pre-medication prior to the beginning of the infusion includes oral paracetamol 1000 mg, intravenous methylprednisolone 250 mg and oral chlorphenamine 4 mg. Serum samples were taken at four time points post-treatment; early (defined as 1–3 months following rituximab), 6, 12 and 36 months following treatment with rituximab (*n* = 114) (see Supplementary Fig. S1 for study flow chart, available at *Rheumatology* online). Inclusion criteria for this study included (i) aged >18 years old at entry to study; (ii) fulfilling the revised 1997 ACR classification for SLE [10]; and (iii) a minimum of 36 months follow-up from entry to the study following rituximab treatment. All patients receiving rituximab were required to have active disease defined as either British Isles Lupus Assessment Group (BILAG) 1A or 2B as per standards of care and national drug commissioning.

### Clinical data

Demographics, concomitant treatment, and laboratory measures including ANA, extractable nuclear antigens (ENA), anti-double stranded DNA (dsDNA) antibodies, CD19^+^ lymphocyte count, immunoglobulin levels (IgG, IgA, IgM), complement C3 (C3) and erythrocyte sedimentation rate (ESR) were collected. Combined clinical and laboratory data was collected at baseline visit (i.e. within 1 month of treatment with rituximab upon entry to this study), early (1–3-), 6-, 12- and 36-month post-treatment time points to match with paired serum samples for assessment of ADA and rituximab level (Supplementary Fig. S1, available at *Rheumatology* online). Follow-up data for all participants was available until December 2019.

### Disease activity and response to treatment

Disease activity was measured using the BILAG-2004 index [11] and Systemic Lupus Erythematosus Disease Activity Index 2000 (SLEDAI-2K) [12]. Global BILAG score was coded as A = 12, B = 8, C = 1 and D/E = 0 [13]. Response to treatment was defined according to BILAG definition and classified as: (i) major response (improvement in all domains rated A/B to C or less and no new A/B flares); (ii) partial response (maximum of one domain with persisting B score with improvement in all other domains and no new A/B flares); (iii) non-response (not fulfilling either major or partial response); and (iv) relapse (new grade A or recurrence of ≥1 B score following a previous major or partial response) as previously described by Yusof *et al.* [14].

### Detection of ADA to rituximab

Two methods were used to evaluate ADAs to rituximab. All samples were analysed using an in-house validated bridging electrochemiluminescent (ECL) immunoassay on the Meso Scale Discovery^®^ (MSD) platform as previously described [2]. To overcome potential drug interference in the bridging ECL assay (particularly in early samples post rituximab infusion) all ADA negative samples with detectable drug level were further analysed using a drug-tolerant precipitation and acid dissociation (PandA) ECL immunoassay, also using the MSD platform. The PandA assay was carried out as previously described by Zoghbi *et al.* [15]. Detailed methodology relating to both techniques can be found in the Supplementary Material, available at *Rheumatology* online.

ADA status of each sample was defined as either negative or positive. Positive samples were further stratified as highly positive (titre ≥16 AU/ml), determined using the upper quartile of the overall cohort’s positive titres. Longitudinally, ADA were defined as persistently positive if they had at least two ADA-positive results within the three years post-treatment with subsequently no negative results. Persistent negative ADA were defined as those with at least two ADA negative tests within the three years post-treatment, with no positive results at any point. All ADA samples were included in cross-sectional analysis. However, those with single samples, fluctuating or transient patterns of ADA were excluded from longitudinal analyses due to limited patient numbers in each group (Supplementary Table S1, available at *Rheumatology* online).

### Measuring serum rituximab level

Serum samples (*n* = 114) were analysed for rituximab concentration using an in-house validated ELISA at the Karolinska University Hospital Immunology Laboratory, Stockholm, Sweden [16]. The ELISA uses an anti-idiotype monoclonal rat antibody against rituximab as the capture reagent and alkaline phosphatase-conjugated goat anti-human IgG ab for detection. The assay’s limits of detection are 0.5–100 µg/ml.

### Evaluating neutralizing capacity of antibodies to rituximab

ADA-positive samples with sufficient sera remaining and undetectable rituximab levels were analysed for neutralizing capacity of ADAs to rituximab (*n* = 38 from 18 patients) using an *in*-*vitro* complement dependent cytotoxicity (CDC) assay as previously described [17], at the Immunology Unit, University Cote d’Azur Hospital, Nice, France. Detailed methodology can be found in the Supplementary Material, available at *Rheumatology* online. Results were reported as either neutralizing (<40% cytotoxicity in the presence of 50 ng/ml rituximab) or non-neutralizing (>70% cytotoxicity in the presence of 50 ng/ml rituximab) for each patient sample. Detailed methodology for this assay can be found in the Supplementary Material, available at *Rheumatology* online.

### Patient involvement

Through our previous extensive patient engagement and involvement events we sought to identify the priorities of patients with SLE with a focus on future research studies. Several key concerns were raised around the tolerability and efficacy of therapy (in particular, rituximab). This was initially raised in a series of focus groups that were held at University College London, UK, where 76.9% of those in attendance reported concerns prior to starting new therapy, with poor response to biologic therapy highlighted as a significant worry [18]. As a result, this current study was designed in an attempt to gain greater understanding into why patients may respond variably to B-cell depletion therapy in clinical practice.

### Statistical analysis

Categorical variables were analysed using Chi-squared or Fisher’s extract test (when sample size <5). Correlation was analysed using Spearman or Pearson’s (depending upon normality of distribution, as assessed by Kolmogorov–Smirnov test). Comparison between persistent ADA-positive and ADA-negative groups was by *t*-test or Mann–Whitney test, while paired t-tests were used for comparisons between variables within the same patient at each time point. All samples (including single positive/negative ADA samples) were included in cross-sectional analysis, while patients were grouped according to persistent positive/persistent negative ADA patterns for longitudinal analysis. Logistic regression was used to identify risk of persistent ADA positivity adjusted for age at diagnosis, cumulative rituximab dose at entry to study, baseline SLEDAI-2K and Global BILAG score. Sex and ethnicity were excluded due to the predominance of females and too few cases in each ethnic group. Statistical significance was defined as *P*-value <0.05. Analyses were completed using R (4.1.10) and GraphPad Prism (9.1.0).

### Ethical approval

This study was approved by the London-Harrow Research Ethics Committee (Ref 11/LO/0330), South Central – Hampshire B Research Committee B (Ref 14/SC/1200) and the Stockholm Regional Ethics Committee (Ref 2019–04420). It was conducted in accordance with the Declaration of Helsinki and all patients provided written informed consent on entry into the study.

## Results

### ADA are common, persistent, and detected both earlier and at higher titre in those previously treated with rituximab

In total, 114 samples from 35 patients with SLE were collected for analysis. This included 19 rituximab-niave patients (54%) undergoing treatment for the first time at entry to the study. Whilst not an exclusion criterion, no patients received concurrent cyclophosphamide or belimumab at time of treatment with rituximab. The remaining 16 patients (46%) having been treated with rituximab prior to the cycle given at entry to this study (median 1 previous cycle, IQR 2–3, range 1–3) (Supplementary Table S2, available at *Rheumatology* online). Over the 36 months of follow-up, 11 patients included in this study required retreatment with another cycle of rituximab due to further flare of the disease.

Baseline demographics and clinical characteristics are summarized in Table 1. There was no observed difference in disease activity at entry into the study between those who were subsequently persistently ADA positive compared with persistently ADA negative in terms of both SLEDAI-2K and Global BILAG score. The most common manifestations at time of treatment included renal involvement (48.6%), rash (34.3%), alopecia (22.9%) and inflammatory arthritis (14.3%). Of the 35 patients included in the study, ADA were frequent with 26 patients (74%) found to be positive at least once during the 36-month follow-up period, of which 18/26 were persistently positive and 12/26 were persistently high positive. Nine patients were persistently ADA negative.

**Table 1.** Patient demographics and clinical characteristics at study baseline infusion

	Persistent positive (*n* = 18)		Persistent negative (*n* 7)	Total cohort^a^
	All persistent positive (*n* = 18)	Persistent high positive (*n* = 12)		
Sex, % female (*n*)	83 (15)	75 (9)	100 (7)	91.4 (32)
Age at diagnosis, median years (IQR)	23.5 (14.25–30.25)	20.5 (12.75–28.50)	38.0 (27.0-48.0)	25 (15–32)
Age at entry to study, median years (IQR)	35.08 (29.17–49.46)	35.00 (29.17–52.33)	51.25 (32.67–53.67)	35.35 (29.17–50.33)
Disease duration at entry to study, median years (IQR)	16 (8.27–19.23)	14.67 (6.97–18.88)	7 (5.92–10.08)	10.9 (6–16.7)
Ethnicity				
Afro-Caribbean, % (*n*)	33.3 (6)	41.67 (5)	42.9 (3)	34.28 (12)
South Asian, % (*n*)	22.2 (4)	16.7 (2)	14.2 (1)	17.14 (6)
Caucasian, % (*n*)	38.9 (7)	33 (4)	28.5 (2)	37.14 (13)
East Asian, % (*n*)	5.6 (1)	8.3 (1)	14.2 (1)	5.71 (2)
Auto-antibodies				
ANA, % positive (*n*)	88.9 (16)	91.6 (11)	85.7 (6)	91.4 (32)
Anti-Sm, % positive (*n*)	27.8 (5)	50 (6)	28.5 (2)	25.7 (9)
Anti-RNP, % positive (*n*)	55.6 (10)	58.3 (7)	42.8 (3)	45.7 (16)
Anti-Ro, % positive (*n*)	50 (9)	50 (6)	57.1 (4)	51.4 (18)
Anti-La, % positive (*n*)	0	0	14.2 (1)	8.57 (3)
Anti-dsDNA, median IU/ml (IQR)	99 (29.5–450.5)	167.5 (26.5–640)	59 (9–506.3)	92 (20–422)
Concomitant medications				
Prednisolone dose, median mg (IQR)	9 (5–16.25)	6.25 (5–13.75)	10 (7–12.5)	10 (5–15)
Prednisolone, % (*n*)	88.9 (16)	91.67 (11)	85.7 (6)	88.5 (31)
Hydroxychloroquine, % (*n*)	50 (9)	41.7 (5)	57.1 (4)	48.5 (17)
Azathioprine, % (*n*)	16.6 (3)	16.7 (2)	0	11.4 (4)
Mycophenolate mofetil, % (*n*)	11.1 (2)	16.7 (2)	0	8.5 (3)
Methotrexate, % (*n*)	0	0	0	5.7 (2)
Ciclosporin	0	0	0	0
Cyclophosphamide	0	0	0	0
Calcineurin inhibitor	0	0	0	0
ACEi, % (*n*)	22.2 (4)	33 (4)	0	14.3 (5)
ARB, % (*n*)	16.6 (3)	16.7 (2)	0	8.5 (3)
Previously treated with RTX, % (*n*)	50 (9)	42 (5)	43 (3)	43 (15)
Prior number of cycles of RTX in those previously treated, median (IQR)	2 (1–2)	1.5 (1–2)	1 (1–2)	1 (1–2)
Disease characteristics				
Renal involvement, % (*n*)	50 (9)	50 (6)	28.5 (2)	48.6 (17)
Neurological involvement, % (*n*)	0	0	14.2 (1)	2.9 (1)
Cardiac involvement, % (*n*)	0	25 (3)	0	0
Respiratory involvement, % (*n*)	0	0	0	0
Alopecia, % (*n*)	22.2 (4)	25 (3)	14.2 (1)	22.9 (8)
Arthritis, % (*n*)	16.6 (3)	8.3 (1)	14.2 (1)	14.3 (5)
Arthralgia, % (*n*)	16.6 (3)	16.7 (2)	42.8 (3)	28.6 (10)
Rash, % (*n*)	33.3 (6)	33 (4)	57.1 (4)	34.3 (12)
Vasculitis, % (*n*)	11.1 (2)	16.7 (2)	0	86 (3)
Mucocutaneous, % (*n*)	11.1 (2)	8.3 (1)	14.2 (1)	11.4 (4)
Myositis, % (*n*)	0	0	0	0
Fevers, % (*n*)	0	0	14.2 (1)	5.7 (2)
Haematological involvement, % (*n*)	0	0	0	0
Disease activity				
SLEDAI-2K, mean (s.d.)	10.67 (4.45)	10.83 (4.71)	11.29 (4.61)	10.73 (4.42)
Global BILAG Score, mean (s.d.)	13.06 (6.17)	13.75 (6.74)	15.14 (7.90)	14.88 (7.16)

a All patients (*n* = 35) are included in the total cohort description. ADA categories excluded from table due to *n* ≤ 5 per group include transient/fluctuating ADA positive *n* = 3, single ADA-positive *n* = 5 and single ADA-negative *n* = 2.

ACEi: angiotensin-converting-enzyme inhibitors; ADA: anti-drug antibody; ARB: angiotensin receptor blockers; dsDNA: double stranded DNA; IQR: interquartile range; *n*: number; RNP: ribonucleoprotein; Sm: Smith; SLEDAI-2K: SLEDAI 2000.

**Figure 1.**
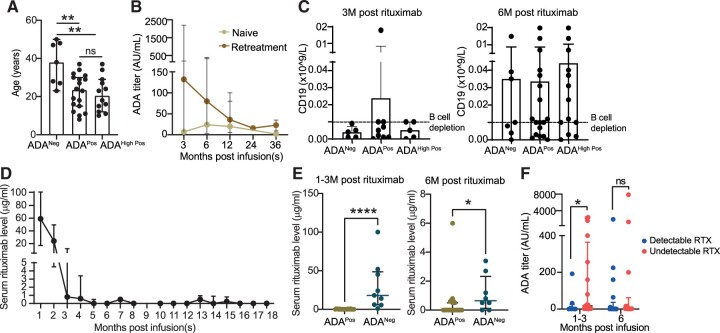
ADA titres peak earlier and at higher titres in patients with previous exposure to rituximab and are associated with lower serum drug levels. **(A)** ADA negative patients were younger than ADA persistently positive and persistently high ADA positive patients (one-way ANOVA and Tukey’s multiple comparisons test, *P* = 0.003). **(B)** Difference in ADA titre comparing those previously treated with rituximab *vs* rituximab naive. **(C)** CD19^+^ lymphocyte count showed no difference between ADA negative, positive and high positive at three months (one-way ANOVA *P* = 0.821) and six months post-treatment (*P* = 0.854). **(D)** Serum rituximab levels (*n* = 114 samples) over time. **(E)** ADA-positive patients had significantly lower serum rituximab levels measured at 1–3 months (Mann–Whitney, *P* < 0.0001) and 6 months post infusion (*P* = 0.018). **(F)** Differences in ADA titres in patients with undetectable and detectable serum rituximab at 1–3 months and 6 months post infusion (Mann–Whitney, *P* = 0.05). ADA: anti-drug antibody; ADA^Neg^: ADA persistent negative; ADA^Pos^: ADA persistently positive; ADA^High pos^: ADA titre >16AU/mL; IQR: interquartile range; ns: not significant. *P < 0.05; **P < 0.01; ****P < 0.0001

Patients who were persistently ADA positive had a significantly younger age of diagnosis of SLE than those who were persistently ADA negative [mean 22.50 (9.10) *vs* 37.29 (11.31) years, *P* = 0.002, Fig. 1A and Table 1]. Logistic regression demonstrated that older age at diagnosis was associated with reduced incidence of persistent ADA formation with a 22% decrease in risk for each addition year after diagnosis (*P* = 0.03, Supplementary Table S3, available at *Rheumatology* online).

### ADA to rituximab is associated with lower serum drug levels and increased ADA neutralizing capacity

In patients who were rituximab niave at entry to the study, ADA were first detected at mean 6.44 [1.10] months post-treatment, except for one patient who developed ADA at 12 months following treatment (having been ADA negative when assessed at 6 months). After peaking at six months post-treatment (ADA titre median 24, IQR 2–328 AU/ml) they gradually waned over the 36-month follow-up period. In comparison, ADA were detected earlier in patients who had previously been treated with rituximab prior to entry to the study [mean 3.18 (0.65) months, *P* < 0.0001] and were also found at higher titres (median 132 AU/ml, IQR 2–2200 at 1–3 months). ADA also decreased over time in those previously treated with rituximab but remained higher overall when compared with those who were treatment naive during the 36-month follow-up period (Fig. 1B).

There was no difference in CD19^+^ lymphocyte count at the early (1–3) and six-month (Fig. 1C) time points between ADA persistently positive and negative patients. Serum rituximab drug levels were also detectable in 42/114 samples (19 patients). In most patients, serum drug levels peaked at 1–3 months waning to an undetectable level at six months (Fig. 1D). However, at both three- and six-months post-treatment, patients with positive ADA had significantly lower serum rituximab levels compared with those who were ADA negative (Fig. 1E). Furthermore, significantly higher ADA titres were seen in samples with undetectable drug level at three months (*P* = 0.030) post infusion (Fig. 1F). No difference was observed at six months post infusion (*P* = 0.55).

Eighteen ADA-positive patients (38 serum samples) had undetectable drug levels, therefore, the neutralizing capacity of these ADA was assessed. A total of 24 samples from 10 patients (55.6%) had neutralizing ADAs with <40% cytotoxicity in the presence of 50 ng/ml rituximab (median ADA titre 175 AU/ml; IQR 80–820; range 6–2400 AU/ml). The neutralizing ADA status of the remaining 14 samples from eight patients (44.4%) could not be reliably determined due to complement dependent B-cell cytotoxicity of the patient sera in the absence of rituximab, with no change in cytotoxicity observed increasing concentrations of rituximab (data not shown). Of these, six samples were borderline (cytotoxicity 40–60%) and eight samples (patients = 8) were cytotoxic (>70%) in the absence of rituximab (Supplementary Fig. S2, available at *Rheumatology* online).

### ADA to rituximab were associated with increased risk of relapse

**Figure 2.**
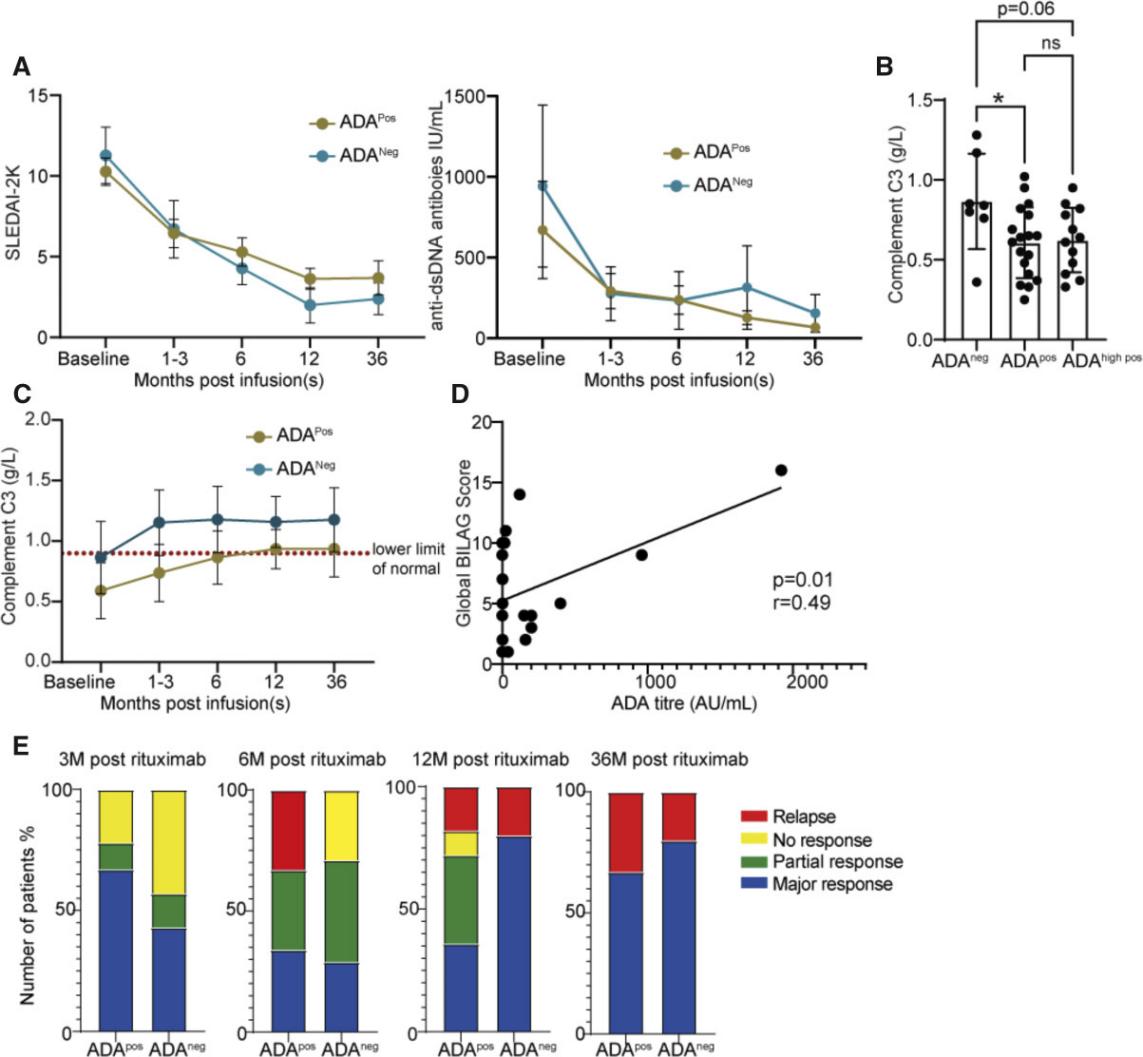
ADA positivity correlates with disease activity at 6 months post-treatment and shows delayed normalization of C3 levels. **(A)** Longitudinal analysis of SLEDAI-2K and anti-dsDNA. **(B)** Baseline Completement C3 levels between ADA negative, ADA positive and ADA high positive (one-way ANOVA and Tukey’s multiple comparisons test, *P* = 0.049). **(C)** Longitudinal analysis of C3 levels over time, from baseline to 36 months in ADA-positive and ADA-negative patients, mean (s.d.). *t*-tests between ADA positive *vs* ADA negative at 1–3 months, *P* = 0.002; 6 months, *P* = 0.004; 12 months, *P* = 0.018; and 36 months, *P* = 0.101, post-treatment. **(D)** Correlation with Global BILAG score *vs* ADA titre at six months (Pearson correlation, r = 0.49, *P* = 0.01). **(E)** BILAG response comparing ADA persistently positive and persistently negative patients. ADA: anti-drug antibody; ADA^Neg^: ADA persistent negative; ADA^Pos^: ADA persistently positive; ADA^High pos^: ADA titre >16AU/mL; dsDNA: double stranded DNA; IQR: interquartile range; ns: not significant; SLEDAI-2K: SLEDAI 2000. *P < 0.05; **P < 0.01; ****P < 0.0001

As expected, a reduction in SLEDAI-2K and anti-dsDNA antibody levels was seen in both persistent ADA-positive and ADA-negative patients at all timepoints following rituximab treatment (Fig. 2A; Supplementary Fig. S3A/B, available at *Rheumatology* online), likely reflecting the known effect of treatment on serological markers of disease activity. Complement C3 level was the only serological marker significantly differentially expressed between ADA-positive and ADA-negative patient groups. SLE patients with persistently positive ADA to rituximab had a significantly lower C3 at baseline when compared with ADA negative patients [mean 0.61 (0.23) g/l *vs* 0.87 (0.30) g/l, *P* = 0.026, Fig. 2B]. C3 levels remained lower in ADA positive compared with ADA negative patients up to 12 months post-treatment (Fig. 2C; Supplementary Fig. S3C, available at *Rheumatology* online). It was also noted that a statistically significant increase in C3 level was reached earlier in those who were ADA negative (within 1–3 months, Wilcoxon *P* = 0.031) but not in those who were ADA positive (paired *t*-test *P* = 0.128), who instead showed a significant improvement at six months post-treatment (paired *t*-test *P* < 0.001).

Interestingly, using the BILAG disease activity index, which is more weighted towards clinical rather than serological assessment of disease activity, a positive correlation was observed between Global BILAG and ADA titre at six months (*P* = 0.01, r = 0.49, Fig. 2D). More detailed analysis found that patients who were persistently ADA positive had either a partial or complete response by the early time point (3 months) but then had higher rates of relapse at six months post-treatment (Fig. 2E). These relapses were typically within the same organ domain with recurrence of renal activity (*n* = 2), haematological disease (*n* = 3) and skin flare (*n* = 1) observed. By 12 months post-treatment, those who were persistently ADA negative showed a higher proportion of major BILAG responders and by 36 months post-treatment there was no significant difference seen (Fig. 2E). Cases of relapse at 36 months post-treatment all related to new disease activity within domains that were previously not active at entry to this study. A summary of clinical response and markers of disease activity at each time point is summarized in Table 2. This suggests that the presence of ADA to rituximab is associated with increased risk of BILAG relapse when assessing clinical response to treatment.

**Table 2.** Long-term clinical outcomes

	Time point following RTX treatment	1–3 months			6 months			12 months			36 months		
		Persistent positive	Persistent high positive	Persistent negative	Persistent positive	Persistent high positive	Persistent negative	Persistent positive	Persistent high positive	Persistent negative	Persistent positive	Persistent high positive	Persistent negative
	Total patients, *n*	15	9	7	18	12	7	16	11	5	13	9	5
**Laboratory markers**	ESR	13 (10–29)	20 (9–43)	21 (8–24)	28 (14–39)	28 (16–48)	28 (18–30)	21 (8–37)	26 (8–38)	15 (8–39)	7 (6–11)	7 (6–11)	16 (11–31)
	C3	0.78 (0.24)	0.84 (0.26)	1.15 (0.27)	0.86 (0.20)	0.91 (0.19)	1.18 (0.27)	0.90 (0.17)	0.90 (0.17)	1.16 (0.21)	0.94 (0.24)	0.94 (0.28)	1.18 (0.26)
	Δ C3	0.17 (0.30)	0.20 (0.37)	0.20 (0.23)	0.25 (0.25	0.28 (0.27)	0.31 (0.29)	0.29 (0.26)	0.30 (0.28)	0.34 (0.28)	0.39 (0.28)	0.37 (0.28)	0.37 (0.39)
	dsDNA ab titre	61 (27–201)	53 (18–572)	98 (9–308)	45 (24–292)	45 (13–347)	45 (8–424)	39 (15–291)	20 (15–422)	31 (9–765)	40 (18–141)	37 (12–174)	28 (1–374)
	Δ dsDNA ab titre	–461 (1439)	–732 (1847)	–666 (1098)	–417 (1364)	–631 (1679)	–317 (493)	–588 (1840)	–819 (2210)	–976 (1340)	–823 (2103)	–1180 (2552)	–1137 (1369)
	CD19 count, median (IQR)	0.011 (0.04)	0.018 (0.08)	0.010 (0.04)	0.034 (0.05)	0.044 (0.06)	0.035 (0.05)						
	Complete B cell depletion, %	44.4%	36.3%	57%	33%	23%	43%						
**Disease activity**	SLEDAI-2K	5.73 (2.92)	5.55 (1.94)	6.71 (4.72)	5.00 (3.96)	5.00 (3.67)	4.29 (2.69)	3.94 (3.20)	4.20 (2.28)	2.00 (2.45)	3.23 (3.61)	3.33 (3.87)	2.40 (2.19)
	Δ SLEDAI-2K	–5.20 (4.89)	–5.78 (5.04)	–4.57 (5.77)	–5.67 (5.05)	–5.83 (5.62)	–7.00 (5.60)	–7.19 (4.67)	–7.46 (5.22)	–10.80 (6.90)	–7.54 (3.93)	–7.33 (4.47)	–10.40 (6.43)
	Global BILAG Score	5.27 (3.26)	4.22 (3.11)	8.14 (7.38)	6.06 (4.05)	6.58 (4.48)	7.71 (5.47)	6.69 (7.21)	5.82 (12.07)	3.80 (3.03)	3.62 (4.29)	4.22 (5.12)	3.20 (2.49)
	Δ Global BILAG Score	–8.07 (8.12)	–10.22 (5.60)	–7.00 (11.8)	–7.00 (6.83)	–7.17 (7.25)	–7.43 (4.39)	–7.25 (9.36)	–4.60 (11.15)	–13.8 (10.43)	–9.69 (7.64)	–9.33 (9.04)	–14.40 (9.79)
	Major BILAG Response, *n* (%)	8 (54)	6 (67)	3 (43)	7 (39)	4 (33)	2 (29)	7 (44)	4 (36)	4 (80)	10 (77)	6 (67)	4 (80)
	Partial BILAG Response, *n* (%)	2 (13)	1 (11)	1 (14)	6 (33)	4 (33)	3 (43)	5 (31)	4 (36)	0 0	0 0	0 0	0 0
	No BILAG Response, *n* (%)	5 (33)	2 (22)	3 (43)	1 (6)	0 0	2 (29)	1 (6)	1 (10)	0 0	0 0	0 0	0 0
	Relapse BILAG Response, *n* (%)				4 (22)	4 (33)	0 0	3 (19)	2 (18)	1 (20)	3 (23)	3 (33)	1 (20)

Values expressed as median (IQR) or mean (s.d.) depending upon normality of distribution. BCC: CD19^+^ B-cell count; BCD: B-cell depletion; BILAG: British Isles Lupus Assessment Group; C3: complement C3; CRP: C-reactive protein; dsDNA: double stranded DNA; ESR: erythrocyte sedimentation rate; SLEDAI-2K: Systemic Lupus Erythematosus Disease Activity Index 2000. Total patients in each category vary as patients with missing clinical data are excluded from the respective time point.

## Discussion

In this study, we demonstrate dynamics of ADA to rituximab over long-term follow-up in a cohort of patients undergoing treatment for SLE. This study also confirms that ADA are highly prevalent in those who received treatment for SLE that has been observed previously [14, 19, 20], with 64.3% of patients persistently positive over the 36-month follow-up period. Furthermore, nearly half of those found to be ADA positive in this study (46%) had persistent high ADA titres (>16 AU/ml). In addition, we found that ADA titres peaked earlier and at high titres in those patients who had previously been exposed to rituximab when compared with those who were previously treatment naive. This may be explained by B- and T-cell memory following prior drug immunization [21–23]. Although antibody isotypes were not determined in this study, this finding would support the notion that re-exposure to rituximab in ADA-positive patients can stimulate immunological memory to the drug with generation of a sustained high affinity IgG response.

We confirm our previous findings that patients who are younger at the time of diagnosis of SLE are of greater risk of developing ADA [8, 9, 22]. This may suggest that a potential risk for persistent ADA positivity could relate to a more severe disease phenotype, which is more often seen in patients diagnosed at a younger age [24–26]. In addition, we also observed that those who were ADA positive had a lower baseline C3 level and that these levels remained persistently lower than those seen in ADA-negative patients over the first 12 months following treatment with rituximab, which again may support more aggressive disease at the time of treatment as being a potential factor in subsequent ADA development. Furthermore, previously it has been demonstrated that younger patients with SLE more frequently have lower C3 levels when compared with adults [24] and this may also explain the differences observed here. A number of previous studies have also proposed that abnormalities relating to complement activation and immune complex formation could also play a role in inducing ADA formation [27].

Furthermore, we describe changes in the kinetics of circulating drug level and note that elimination of the drug may be related to ADA positivity given the strong association with reduction in circulating serum rituximab levels in those with positive ADA. In terms of clinical outcomes, we also found that in spite of early efficacy of treatment, there was increased rates of relapse in ADA-positive patients. This may also reflect a more severe disease phenotype in certain patients and subsequent need for multiple courses of rituximab treatment predisposing them to the development of stable ADA titres, as well as early drug clearance increasing the risk of subsequent flares. In comparison, a greater proportion of ADA-negative patients maintained a clinical response up to 12 months post-treatment which may reflect a less severe disease course as fewer patients had prior exposure to rituximab compared with the ones who developed ADA. It is unclear as to why patients who were persistently ADA positive demonstrated an early significant response to treatment prior to having an increased risk of premature relapse. There was no evidence of difference in CD19^+^ B-cell count between persistent ADA-positive and ADA-negative groups at 3- and 6-months post-treatment, thus suggesting that relapse is independent of circulating B-cell depletion. It could be theorized that ADA to rituximab may also have a long-lived impact within tissue or nodal centres that potentially results in earlier relapse.

By assessing the neutralizing capacity of ADA to rituximab *in vitro*, we confirmed that this was associated with lower drug levels. These findings support previous studies in which ADA were associated with a lack of response to retreatment (secondary non-response) [14]. An improved response was noted in prior non-responders to rituximab who were retreated with a fully humanized anti-CD20 antibody given that ADA formation is more commonly seen against chimeric monoclonal antibody therapies [28]. This may be a potential future treatment option for patients with known ADA as an alternative to rituximab and has already been observed to be effective in those with previous infusion-related reactions [9, 29].

A limitation of our study is that it is a single-centre study with a small sample size. In addition, we included patients with both juvenile and adult-onset and that we could not control for the effect of concurrent medication on the development of ADA, due to the small sample size and use of various background medications which reflects SLE heterogeneity. None of the patients in this study were taking methotrexate, which has previously been reported to reduce anti-drug antibodies directed against anti-TNF therapy [30, 31]. No patients within this study received concurrent treatment with cyclophosphamide. Therefore, it is not possible to evaluate whether combination therapy with either methotrexate or cyclophosphamide reduces subsequent persistent ADA formation in SLE in this study. However, in our previous cross-sectional study of ADA to rituximab in SLE we included 37 patients (out of a cohort of 57) patients who were treated with concurrent cyclophosphamide along with the standard cycle of rituximab and found no significant difference in risk of subsequent ADA formation. Only two patients within that study were taking methotrexate at the time of treatment with rituximab, both did not develop ADA (although larger numbers would need to be investigated before the protective effect of methotrexate against later ADA formation would need to be conducted for this to be fully examined) [9]. Our results do, however, support our previous findings that there is an increased ADA prevalence in younger patients [9]. Further research to validate the findings of our study, in particular in relation to clinical outcomes are required. If our results are confirmed, then they would support further investigation of clinical benefit of therapeutic drug monitoring in routine clinical practice.

In conclusion, ADA to rituximab frequently occur in those undergoing treatment for SLE. Patients with previous rituximab exposure, a younger age of diagnosis and low baseline C3 had a high prevalence of ADA. In addition, ADA to rituximab were associated with early elimination of the drug as demonstrated by significantly lower circulating drug level in ADA-positive patients. In spite of no difference observed in CD19^+^ B-cell counts, ADA-positive patients appeared to show an early improvement followed by a subsequent loss of clinical response resulting in earlier relapses. We propose that this may be a result of the neutralizing capacity of ADA (which we demonstrate *in vitro*) that in turn produces an earlier clearance of circulating drug, which potentially increases the risk for subsequent clinical relapse. If validated in larger studies, the routine assessment of ADA titre both prior to, and following treatment may better predict response to therapy and could provide an early prompt to change management to either an alternative biologic agent or a fully humanized anti-CD20 treatment.

## Supplementary Material

keac608_Supplementary_Data

## Data Availability

The data that support the findings of this study are available from the corresponding author on request.
