# Supplementary material for: Anti-rituximab antibodies demonstrate neutralizing capacity, associate with lower circulating drug levels and earlier relapse in lupus
Source: Rheumatology (Oxford). 2022 Nov 12;62(7):2601–10. doi: 10.1093/rheumatology/keac608 (PMC10321108; doi:10.1093/rheumatology/keac608)
Supplement: keac608_Supplementary_Data [file keac608_supplementary_data.docx]

**Supplementary Material**

**Anti-rituximab antibodies are shown to have neutralising capacity, associate with lower circulating drug levels and earlier relapse in patients undergoing treatment for systemic lupus erythematosus**

Chris Wincup^1*^, Nicky Dunn^2,3*^, Caroline Ruetsch-Chelli^4,5,6^, Ali Manouchehrinia^2,3^, Nastya Kharlamova^2,3^, Meena Naja^7^, Barbara Seitz-Polski^4,6^, David A Isenberg^1^, Anna Fogdell-Hahn^2,3+^, Coziana Ciurtin^1,7^, Elizabeth C Jury^1+^

^*^Wincup & Dunn co-first authorship,

^+^Fogdell-Hahn, Ciurtin & Jury co-senior authorship

**Affiliations**

^1^Centre for Rheumatology Research, Division of Medicine, University College London, Rayne Building, London WC1E 6JF, United Kingdom

^2^Department of Clinical Neuroscience, Karolinska Institutet, Stockholm, Sweden

^3^Center for Molecular Medicine, Karolinska University Hospital, Stockholm, Sweden

^4^Laboratoire d’Immunologie, CHU de Nice, Université Côte d’Azur, Nice, France

^5^Centre Méditerranéen de Médecine Moléculaire (C3M), INSERM U1065, Université Côte d’Azur, Nice, France

^6^Unité de Recherche Clinique de la Côte d’Azur (UR2CA), Université Côte d’Azur, Nice, France

^7^Centre for Adolescent Rheumatology Research, Division of Medicine, University College London, Rayne Building, London WC1E 6JF, United Kingdom

**Supplemental Methods**

**Detection of ADA to rituximab**

ADA were initially detected using an in-house validated bridging electrochemiluminescent (ECL) immunoassay on the Meso Scale Discovery® (MSD) platform as previously described.^1^ In brief, ADAs present were detecting in a bridging assay using biotinylated and ruthenylated rituximab as capture and reporter molecules. Samples were first screened and if reactive, were analysed in a competitive assay which uses excess unlabelled drug to confirm specificity of ADAs to rituximab. SLE specific screening and confirmation cut points were used with a 5% and 1% false positive rate for screening and confirmation, respectively. Confirmed ADA positive samples were titrated in the third tier to give a titre in arbitrary units per millilitre (AU/mL).

To overcome potential drug interference in the bridging ECL assay (particularly in early samples post rituximab infusion), ADA negative samples with detectable rituximab drug level were further analysed using a drug tolerant precipitation and acid dissociation (PandA) ECL immunoassay, also using the MSD platform. The PandA assay was carried out as previously described by Zoghbi et al., adapted to detect ADA to rituximab.^2^ Briefly, excess rituximab was added to each sample to saturate ADA present and form ADA-rituximab complexes. Complexes were then precipitated using PEG and reconstituted before a final acid treatment step to prevent reformation of complexes. Presence of ADAs to rituximab were detected by adding ruthenylated rituximab as a reporter. Samples analysed using the PandA assay were screened, and if positive, were analysed in a competitive confirmation assay to determine binary status.

**Evaluating neutralising capacity of antibodies to rituximab**

ADA positive samples with sufficient sera remaining and undetectable rituximab levels to avoid drug interference were analysed for neutralizing capacity of ADAs to rituximab (n= 38 from 18 patients) using an in-vitro complement dependent cytotoxicity (CDC) assay as previously described.^3^ In brief, 10 μl of ADA positive patient serum was incubated with 10 μl of rituximab at different concentrations (0 ng/ml, 6 ng/ml, 12.5 ng/ml, 25 ng/ml, and 50 ng/ml) for two hours at room temperature. One microliter of each sample was added in duplicates to 1.5 × 10^3^ purified B-cells (Macs prep HLA B cell isolation kit) from healthy donors with HLA typing by PCR sequence specific oligonucleotid SSO in a 60-well Terasaki plates^4^ and incubation at room temperature for 30 minutes. Five microlitres of standard rabbit complement (Lot 2692) was then added to each well and incubated for an additional 45 minutes at room temperature. Then 2.5 μl Fluoroquench AO/EB staining/quench (Lot SOABATCH 639687) was added to each well for ten minutes in the dark, before two blinded independent evaluators estimated the percentage of dead cells under fluorescent microscope. This experiment was repeated with heat-inactivated serum. ADA positive sera were heated at 56◦C for 30 min to inhibit endogenous complement activity before incubation with rituximab. Sera from healthy donors were used as negative controls, and to prepare positive controls spiked with rat anti-rituximab antibodies. Other controls including anti-lymphocytic serum (Lot 9W2049), AB serum (Lot 59208317290), B+ and anti-pan B antibodies were also used as previously described.^3^ ADA positive samples were reported as either neutralising (<40% cytotoxicity in the presence of 50 ng/ml rituximab) or non-neutralising (>70% cytotoxicity in the presence of 50 ng/ml rituximab).

**Supplementary Results**

**Supplementary Figure S1: Study design flow chart**

**
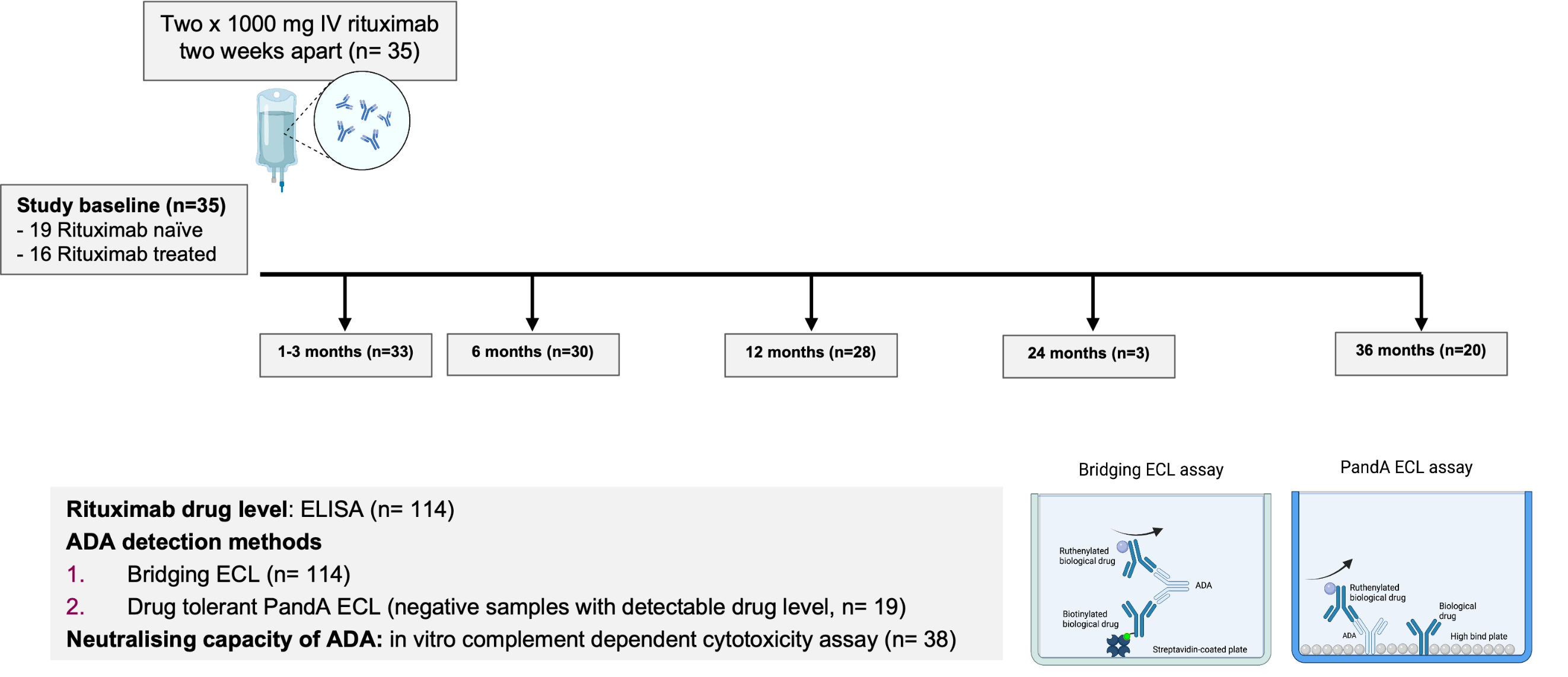
**

Supplementary Figure S1: Study design flow chart demonstrating timeline of included samples, and methods used in the study (IV, intravenously: PandA, precipitation and acid dissociation; ECL, electrochemiluminescent). Figure created in *Biorender.com.*

**Supplementary Table S1. Presence and persistence of ADA in SLE patients (n=35)**

|  | | | | | | | | |
| --- | --- | --- | --- | --- | --- | --- | --- | --- |
|  | **ADA positive** | | | | | **ADA negative** | | **Total Cohort** |
|  | **Persistent low positive** | **Persistent high positive** | **Single positive** | **Transient** | **Fluctuating** | **All persistent negative** | **Single negative** |  |
| Total, % (*n*) | 17.14 (6) | 34.29 (12) | 14.28 (5) | 2.85 (1) | 5.71 (2) | 20 (7) | 5.71 (2) | 100 (35) |
| Samples, % (*n*) | 19.3 (22) | 42.98 (49) | 4.39 (5) | 1.75 (2) | 12.28 (14) | 17.54 (20) | 1.75 (2) | 100 (114) |
| Titre (AU/mL), median (IQR) | 2 (2-2), 2-16 | 80 (16-225), 2-7860 | 32 (14-1920), 4-2240 | 16 (2-160), 0-160 | 2 (2-7), 0-112 | n/a | n/a | n/a |
| ADA, anti-drug antibody; n, number; IQR, Interquartile range | | | | | | | | |

**Supplementary Table S2. Rituximab dose and infusions**

|  | | | | | | | | |
| --- | --- | --- | --- | --- | --- | --- | --- | --- |
|  | **ADA positive** | | | | | **ADA negative** | | **Total Cohort (n=35)** |
|  | **All persistent positive (n=18)** | **Persistent high positive (n=12)** | **Single positive (n=5)** | **Fluctuating (n=2)** | **Transient (n=1)** | **All persistent negative (n=7)** | **Single negative (n=1)** |  |
| First RTX cycle at study baseline, % (*n*) | 56 (10) | 50 (6) | 80 (4) | 50 (1) | 0 (0) | 57 (4) | 0 | 54 (19) |
| RTX cycles prior to study baseline, median (IQR), range | 0 (0-1), 0-2 | 0 (0-1), 0-2 | 0 (0-0.5), 0-1 | 1 (1-1-), 1-2 | 3 | 0 (0-1), 0-2 | 1.5 (1-2), 1-2 | 0 (0-1), 0-3 |
| RTX cycles at end of follow up, median (IQR), range | 2 (1-3), 1-5 | 2 (1.25-2.75), 1-4 | 1 (1-1.5), 1-2 | 3 (2-4), 2 | 5 | 2 (1-2), 1-6 | 2.5 (2.3), 1-3 | 2 (1-3), 1-6 |
| RTX, Rituximab; ADA, anti-drug antibody; n, number; IQR, Interquartile range | | | | | | | | |

**Supplementary Table S3. Logistic regression model**

|  | | | |  |
| --- | --- | --- | --- | --- |
|  | **Persistent positive vs. Persistent negative** | | |  |
| *Predictors* | *Odds Ratios* | *CI* | *p* |  |
| Age at disease diagnosis | 0.78 | 0.55 – 0.92 | **0.033** |  |
| Total rituximab cycles at study baseline | 0.86 | 0.15 – 4.49 | 0.845 |  |
| Baseline global BILAG score | 0.68 | 0.29 – 1.04 | 0.193 |  |
| Baseline SLEDAI-2K score | 1.39 | 0.82 – 3.96 | 0.369 |  |
| Observations | 23 |  |  |  |
| R2 Tjur | 0.498 |  |  |  |
| BILAG, British Isles Lupus Assessment Group; SLEDAI-2K, Systemic Lupus Erythematosus Disease Activity Index 2000; | | | |  |
|  |  |  |  |  |

**Supplementary Figure S2: ADA to rituximab have neutralising capacity**

**Supplementary Figure S2**: The results of *in vitro* analysis for rituximab neutralisation demonstrated ADA to rituximab can have neutralising capacity. Eighteen ADA positive samples (10 patients) were observed to have neutralizing capacity *in-vitro* (< 40% B cell cytotoxicity in the presence of 50 µg/ml rituximab). The neutralising ADA status of the remaining 14 ADA positive samples from eight patients (44.4%) could not be reliably determined due to complement dependent B-cell cytotoxicity of the patient sera in the absence of rituximab (data not shown). The black dotted line reflects the threshold for B-cell cytotoxicity (>70%), while the blue dotted line reflects the absence of cytotoxicity (<40%). RTX, Rituximab; HD, Healthy Donor.

**
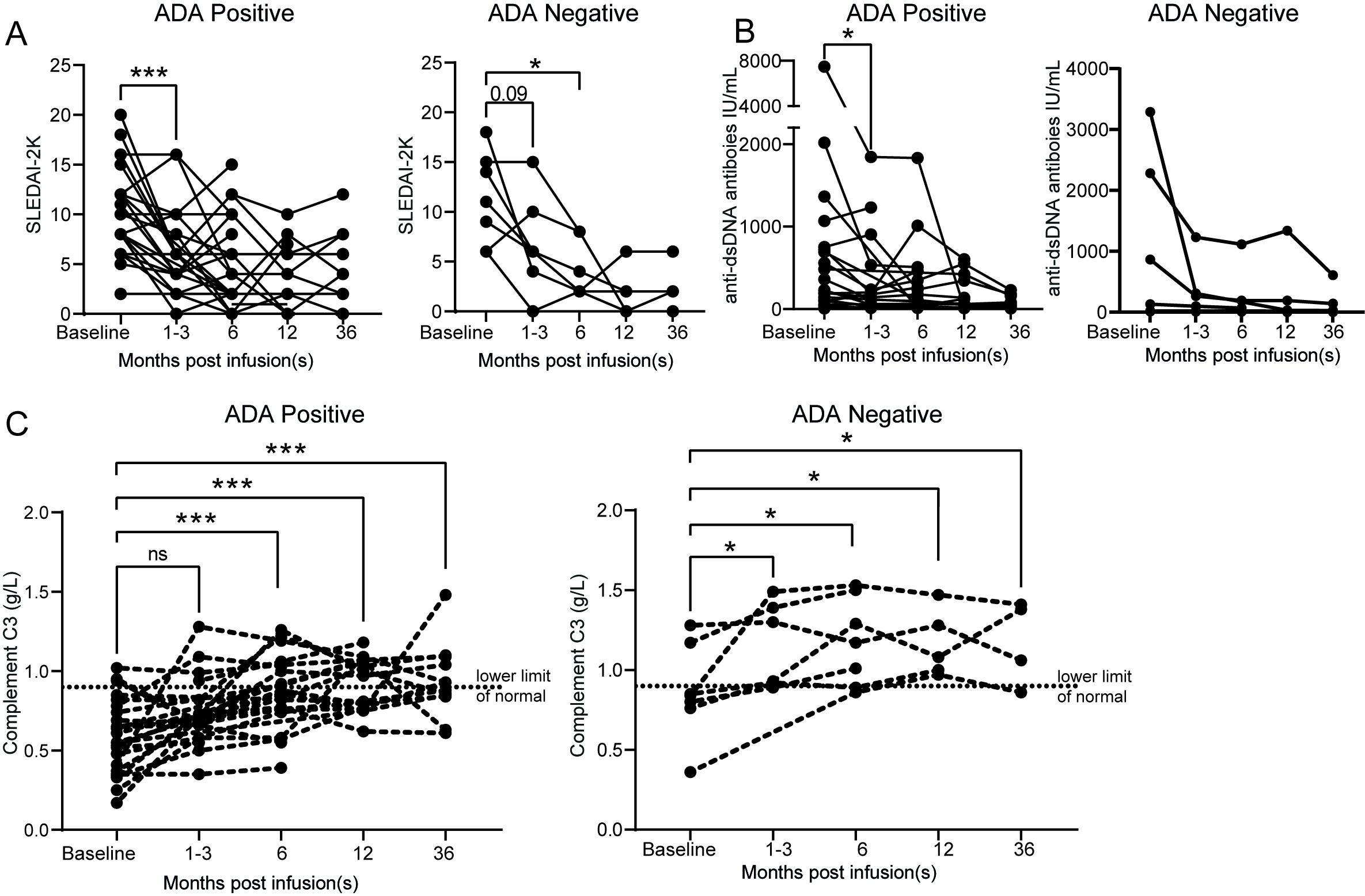
**

**Supplementary Figure S3: ADA positivity is associated with changes in disease activity, dsDNA and complement C3. (A)** In ADA positive and ADA negative patients, SLEDAI-2K improved following treatment at the 1-3 months post-treatment time point. A significant mean reduction in SLEDAI-2K in ADA positive patients of 4.30 ± 4.88 from baseline was observed (n=28, Wilcoxon test p=0.0004). Whilst a mean reduction of SLEDAI-2K in ADA negative patients of 4.57 ± 5.77 was seen at 1-3 months post-treatment this did not reach significance (n=7, paired t-test p=0.09) at this time point but was significant at six months post-treatment (paired t-test p=0.016). (**B).** There was no significant difference in baseline anti-dsDNA antibody titres at baseline (ADA positive n=25, median 189, IQR 48-706 IU/ml vs ADA negative n=7, median 133, IQR 11-2282 IU/ml, Mann Whitney p>0.999). A reduction in anti-dsDNA antibody titres was observed in both ADA positive (Wilcoxon p=0.037) and ADA negative groups (Wilcoxon p=0.0156) following treatment with rituximab at 1-3 months. **(C)** In addition to having lower baseline C3 levels, ADA positive patients had persistently lower C3 levels compared with ADA negative at 1-3- (mean 0.74 ± 0.24 g/L vs mean 1.15 ± 0.27 g/L, t-test p=0.002), 6- (mean 0.87 ± 0.22 g/L vs mean 1.18 ± 0.27 g/L, t-test p=0.004) and 12- months (mean 0.94 ± 0.16 g/L vs mean 1.16 ± 0.21 g/L, t-test p=0.018) following treatment with rituximab. There was no significant difference in C3 between ADA positive and negative patients at 36 months post-rituximab (mean 0.94 ± 0.23 g/L vs mean 1.18 ± 0.26 g/L, t-test p=0.101).

**Supplementary file references**

1. Dunn N, Juto A, Ryner M, Manouchehrinia A, Piccoli L, Fink K, et al. Rituximab in multiple sclerosis: Frequency and clinical relevance of anti-drug antibodies. Mult Scler. 2018;24(9):1224-33
2. Zoghbi J, Xu Y, Grabert R, Theobald V, Richards S. A breakthrough novel method to resolve the drug and target interference problem in immunogenicity assays. J Immunol Methods. 2015;426:62-9.
3. Boyer-Suavet S, Andreani M, Lateb M, et al. Neutralizing anti-rituximab antibodies and relapse in membranous nephropathy treated with rituximab. Front Immunol 2020; 10: 3069.
4. Terasaki P, McClelland P. Microdroplet Assay of Human Serum Cytotoxins. Nature 1964;204:998-1000
